# Ketone monoester intake attenuates exercise performance decline in normobaric hypoxia: A post‐hoc subgroup analysis of 5 young male adults without acute mountain sickness

**DOI:** 10.14814/phy2.71136

**Published:** 2026-10-27

**Authors:** Myrthe Stalmans, Domen Tominec, Wout Lauriks, Ruben Robberechts, Monique Ramaekers, Tadej Debevec, Chiel Poffé

**Affiliations:** ^1^ Exercise Physiology Research Group, Department of Movement Sciences KU Leuven Leuven Belgium; ^2^ Department of Cellular and Physiological Sciences, Faculty of Medicine University of British Columbia Kelowna British Columbia Canada; ^3^ Faculty of Sport University of Ljubljana Ljubljana Slovenia; ^4^ REVAL—Rehabilitation Research Center, Faculty of Rehabilitation Sciences Hasselt University Diepenbeek Belgium; ^5^ Department of Automatics, Biocybernetics and Robotics Jožef Stefan Institute Ljubljana Slovenia

**Keywords:** cognitive function, endurance performance, exogenous ketosis, hypoxia, sleep quality

## Abstract

In hypoxia, endurance performance, sleep, and cognitive function are impaired. Ketone ester (KE) intake can elevate blood, brain, and skeletal muscle oxygenation, making it an interesting strategy to attenuate hypoxia‐induced impairments. Given that acute mountain sickness (AMS) influences these parameters, we evaluated the effects of KE intake in non‐AMS developing individuals. In a larger project, 14 young, healthy, male participants performed a 29‐h protocol at a simulated altitude of 4000–4500 m, involving regular KE or placebo intake. Five participants completed both sessions without developing severe AMS. They completed two maximal incremental cycling tests after 1.5 and 25 h (EX_MAX1_ and EX_MAX2_, resp.) and a combined maximal‐submaximal test after 26.5 h (EX_COMBO_), while resting blood, brain, and skeletal muscle oxygenation and cognitive function were regularly (5‐6x) evaluated, and sleep was analyzed. KE did not alter peak power output (PPO) during EX_MAX1_ or EX_MAX2_, but increased PPO by 36 W during EX_COMBO_. KE increased blood oxygenation before EX_MAX1_, EX_MAX2_, and EX_COMBO_ and increased muscle oxygenation only before EX_COMBO_. Sleep and cognitive function were not affected by KE intake. In a non‐AMS developing subgroup, KE appears effective to attenuate high‐intensity endurance performance decline during subacute hypoxic exposure accompanied by repeated exercise until exhaustion.

## 1 INTRODUCTION

Hypoxic conditions and the resulting drop in blood and tissue oxygenation induce a cascade of adverse physiological effects. Particularly with more severe hypoxia persisting for several hours to days, various functional outcomes are impaired, such as endurance performance (Wehrlin & Hallén, 2006), cognitive function (McMorris et al., 2017; Xu et al., 2025), as well as sleep (Hoshikawa et al., 2007; Selvamurthy et al., 1986) and exercise recovery (Ballmann et al., 2014). Due to the diverse yet impactful challenges induced by hypoxia, there is a continuous search for strategies to improve individuals' hypoxic tolerance. However, identifying and isolating the effect of a given intervention under such severe hypoxic conditions is typically confounded by the occurrence of acute mountain sickness (AMS). AMS is the most prevalent form of altitude illness, with hypoxemia as its most important driver and headache, nausea, dizziness and fatigue as decisive symptoms, and has been shown to transiently exacerbate the decline in exercise performance, cognitive functioning, and sleep quality (Bradwell et al., 1986; Issa et al., 2016; Tang et al., 2014).

In recent years, exogenous ketosis has been extensively studied for its ability to elevate blood and tissue oxygenation (McClure, Phillips, Koutnik, et al., 2024; Poffe et al., 2021; Stalmans et al., 2024; Stalmans, Tominec, Debevec, & Poffé, 2025; Stalmans, Tominec, Lauriks, et al., 2025), proposing ketone supplementation as a particularly interesting candidate to attenuate hypoxia‐induced adverse effects. Indeed, we recently observed that oral ketone ester (KE) intake attenuated AMS development in young, healthy individuals at a simulated altitude of 4000 m (Stalmans et al., 2024). Nonetheless, KE only increases blood and/or tissue oxygenation upon exposure to relatively high altitudes (≥4000 m) (Stalmans, Narang, et al., 2026; Stalmans, Tominec, Narang, et al., 2026; Stalmans, Tominec, Lauriks, et al., 2025; Stalmans, Tominec, Robberechts, et al., 2025). More specifically, KE‐induced acidosis increases ventilation, resulting in a higher partial pressure of oxygen (pO_2_) in the blood. Given the sigmoidal curve of the oxyhemoglobin dissociation curve, such relatively small pO_2_ increase will only increase blood oxygen saturation (SpO_2_) and total oxygen content (ctO_2_) within the steep range of the oxyhemoglobin dissociation curve, encountered with lower baseline pO_2_ levels. Moreover, this effect has been documented to appear gradually rather than instantaneously, requiring >3–4 h to fully establish (Poffe et al., 2021; Stalmans et al., 2024).

Despite the beneficial effect on AMS, earlier studies did not reveal beneficial functional outcomes upon KE during relatively low hypoxic doses (i.e., shorter durations and/or lower altitudes), suggesting that the primary and sole recommended use for acute ketone administration is to mitigate AMS. Indeed, an earlier report from our group (Poffe et al., 2021) revealed that KE intake did not affect 15‐min time‐trial power output after 3 h at a simulated altitude of 3000 m and peak power output during an incremental cycling test was not affected by KE intake after ~70 h at terrestrial altitude (Tominec et al., 2025), while other research demonstrated a 10‐W reduction in peak power output achieved during a ramp protocol after 1.5 h at 4000 m simulated altitude (Stalmans, Tominec, Lauriks, et al., 2025). Similarly, we recently found that KE intake did not affect nocturnal blood saturation, nor sleep quality at a simulated altitude of 3000 m (Stalmans, Tominec, Robberechts, et al., 2025). Nevertheless, KE intake has been shown to mitigate cerebral deoxygenation and hypoxia‐induced disruptions of EEG patterns (Vermaerke et al., 2026), and has been shown to prevent cognitive performance decline at altitudes of ~6100 m (McClure, Phillips, Koutnik, et al., 2024), but not following short exposure to slightly lower altitudes (3000–4500 m) (McClure, Phillips, Kernagis, et al., 2024; Stalmans, Tominec, Lauriks, et al., 2025; Vermaerke et al., 2026).

Therefore, the aim of this study is to explore the potential of KE to improve performance, cognition, and sleep disruptions throughout longer exposure (29 h) to higher altitudes (4000–4500 m). Of particular importance, these effects were evaluated in subjects who did not develop AMS to avoid any potential confounding effect and thereby enable the identification of the true impact of KE on these functional outcomes in hypoxia. Therefore, we analyzed exercise performance, cognitive function, and sleep data collected from five participants who did not develop AMS in either their KE or placebo session following 29 h of exposure to 4000–4500 m simulated altitude in our previous study (Stalmans et al., 2024; Stalmans, Tominec, Lauriks, et al., 2025).

## 2 METHODS

### 2.1 Study design and participants

This research is positioned within a larger project, of which the details have been reported earlier (Stalmans et al., 2024; Stalmans, Tominec, Lauriks, et al., 2025). This project was approved by the Ethics Committee Research UZ/KU Leuven (B3222022000810), preregistered at www.clinicaltrials.gov (NCT05588427), and conducted in accordance with the Declaration of Helsinki guidelines. Fourteen healthy, male participants were recruited for participation in a randomized, double‐blind, placebo‐controlled, crossover study. Despite our intentions to include female participants, the limited availability of the hypoxic testing facility did not allow appropriate, menstrual phase‐based scheduling of the sessions to standardize hormonal balance across the cycle. Hence, only male individuals were included in this study.

The larger study consisted of two 29‐h sessions, during which participants resided at a simulated altitude of 4000 m (F_I_O_2_: 12.7%) for the first 26 h, followed by a prompt transition to 4500 m (F_I_O_2_: 11.8%) for the final 3 h of the protocol, resulting in a total hypoxic exposure of 117,500 m.h per session. A schematic overview of the protocol is illustrated in Figure 1. Throughout both sessions, participants regularly received either ketone ester (KE) or placebo drinks (CON). The protocol was terminated prematurely if the participant developed severe AMS [Lake Louise Score (LLS) of ≥10 out of 15 (Roach et al., 2018)]. No adverse effects other than AMS‐related symptoms were recorded during the study. More information on the termination of participants' session is included in Figure S1. Only five participants completed both sessions without requiring premature protocol termination (Stalmans et al., 2024), and the current work presents data collected throughout the full 29‐h protocol from this subgroup (*n* = 5; age: 25 ± 6 years old; height: 1.86 ± 0.06 m; body mass: 74.6 ± 4.9 kg; peak oxygen consumption rate (V̇O_2_peak): 53.0 ± 4.7 mL.kg^−1^.min^−1^).

**FIGURE 1:**
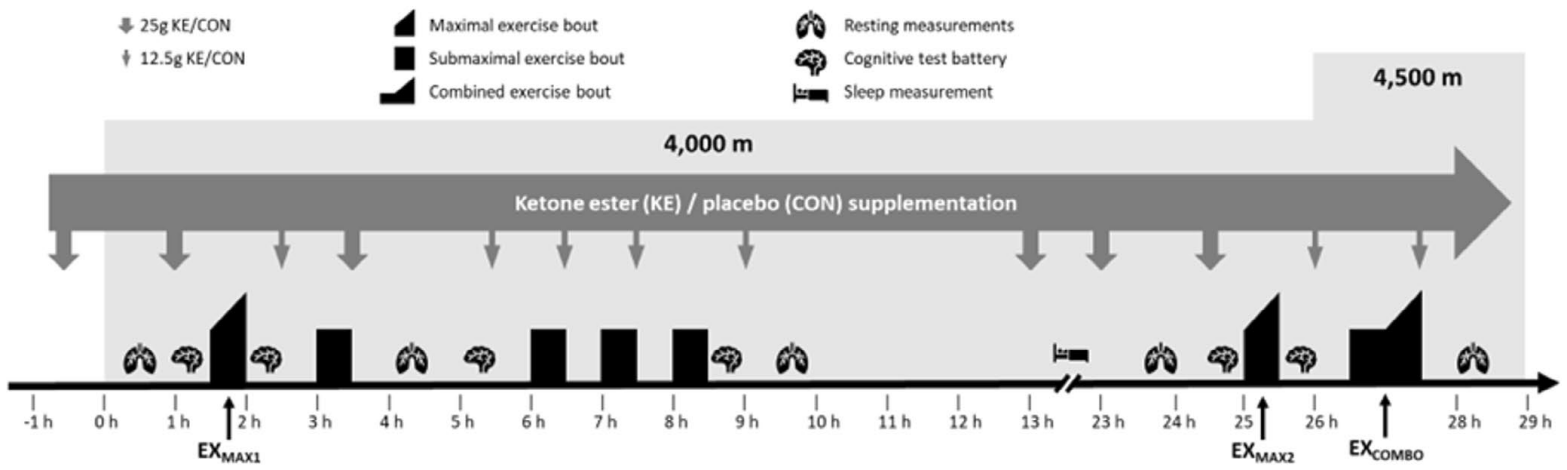
Schematic representation of the experimental sessions. In a double‐blind, randomized, crossover design, 14 participants underwent a 29 h normobaric hypoxic protocol (26 h at 4000 m, followed by 3 h at 4500 m) that was explicitly designed to elicit acute mountain sickness (AMS). Participants were instructed to comply with the protocol, but it was prematurely terminated if they developed severe AMS (Lake Louise score ≥10 out of 15). The present manuscript reports the data collected from the subgroup (*n* = 5) that completed the full protocol in both sessions. Throughout the entire protocol, participants received either ketone ester (KE) or placebo (CON) supplements at regular timepoints through portions of either 25 g (big arrow) or 12.5 g (small arrow). In each session, two maximal (10 min warming up + ramp protocol, EX_MAX1_ and EX_MAX2_), four submaximal (30 min), and a combined exercise bout (30 min + ramp protocol, EX_COMBO_) were completed. After 15 min, as well as 4, 9.5, 23.5, and 28 h hypoxic exposure, participants underwent a physiological characterization at rest, including the determination of blood oxygen saturation, cerebral and muscular oxygenation status, heart rate, and blood pressure. Immediately before and after EX_MAX1_ and EX_MAX2_, as well as after 5 and 8.5 h in hypoxia, participants performed a cognitive test battery. Furthermore, capillary acid–base balance, blood gases, and glucose and lactate concentrations were measured before the onset of EX_MAX1_, EX_MAX2_, and EX_COMBO_.

### 2.2 Familiarization sessions

As detailed in our earlier publication (Stalmans et al., 2024; Stalmans, Tominec, Lauriks, et al., 2025), participants' V̇O_2_peak was determined in normoxia using a protocol consisting of a 10‐min warm‐up at 70 W, followed by a ramp protocol of 100 W ± 10 W every 30 s until voluntary exhaustion. V̇O_2_peak was determined using a Cortex Metalyzer 3b (Cortex, Leipzig, Germany), defined as the highest mean oxygen uptake rate (V̇O_2_) over a 15‐s period. Moreover, they performed two preliminary sessions under normoxic conditions to get familiarized with all measurements and protocols employed in the experimental sessions.

### 2.3 Exercise bouts

Throughout the protocol, participants performed an incremental exercise test until exhaustion after 1.5 and 25 h at altitude (EX_MAX1_ and EX_MAX2_, resp.) to determine their peak power output (PPO) and V̇O_2_peak on a cycling ergometer (Avantronic Cyclus II, Leipzig, Germany). After 10 min of warming up at 1.5 W·kg^−1^ body weight, resistance was set at 100 W and increased by 10 W every 30 s until voluntary exhaustion. Moreover, a 30‐min submaximal exercise bout at a constant power output of 1.5 W·kg^−1^ was performed after 3, 6, 7, and 8 h at altitude (EX_SUBM1_ to EX_SUBM4_, resp.). Finally, after 26.5 h at altitude, participants performed a combined exercise bout (EX_COMBO_) consisting of 30 min at 1.5 W·kg^−1^ immediately followed by 100 W + 10 W every 30 s until volitional exhaustion.

### 2.4 Nutritional intervention

In their KE session, participants received the ketone monoester (R)‐3‐hydroxybutyl (R)‐3‐hydroxybutyrate (KetoneAid Inc., Falls Church, Virginia, USA; ~4.7 kcal.g^−1^) in boluses of either 25 g (30 min before entering hypoxia, as well as after 1, 4, 23, and 24.5 h in hypoxia and 30 min before sleep) or 12.5 g (after 2.5, 5, 6.5, 7.5, 9, 26, and 27.5 h in hypoxia). This supplementation protocol was designed with the purpose to establish a stable ketosis ([ßHB] ~1.5–3 mM). In their CON session, participants received identical boluses of an inert, non‐caloric placebo drink. Dietary intake was standardized from the evening before until the end of the experimental protocol, and generally included carbohydrate‐rich foods (~60% carbohydrate, ~20% fat, ~20% protein; ~4000 kcal during the 29 h protocol excluding KE/CON supplements). Details about the full supplementation strategy and dietary control were described in detail in our earlier publication (Stalmans et al., 2024).

### 2.5 Physiological measurements

Full details of the experimental measurements were reported in our earlier publications from the larger project (Stalmans et al., 2024; Stalmans, Tominec, Lauriks, et al., 2025). An overview of the measurements included in the current manuscript is provided below. After 15 min, and after 4, 9.5, 23.5, and 28 h hypoxic exposure, as well as before the onset of each exercise bout, participants underwent a physiological characterization at rest, including the determination of blood oxygen saturation (SpO_2_) using a pulse oximeter (Nellcor PM10N, Medtronic, Minneapolis), cerebral (prefrontal cortex) and skeletal muscle (*m. vastus lateralis*) tissue oxygenation index (cTOI and mTOI, resp.) using near‐infrared spectroscopy (NIRS, NIRO‐200 spectrometer, Hamamatsu, Japan), mean arterial blood pressure (MAP, Omron M6, Omron healthcare, Kyoto, Japan), and heart rate (HR, Polar H10, Polar, Kempele, Finland). Cognitive function was evaluated immediately before and after MAX1 and MAX2 using a cognitive test battery (Cantab, Cambridge Cognition, Cambridge, UK), as well as after 5 and 8.5 h in hypoxia, to evaluate (i) reaction time (RTI), (ii) rapid visual information processing (RVP), and (iii) spatial working memory (SWM). Outcome parameters of each test were (i) RTI: median reaction time (RTI‐MRT), median movement time (RTI‐MMT), and number of errors out of 30 (RTI‐E); (ii) RVP: mean response latency (RVP‐MRL), number of correct responses (hits) out of 54 (RVP‐H), and false alarms out of 54 (RVP‐FA), and (iii) SWM: strategy score (SWM‐SS) and number of errors (SWM‐E). Capillary D‐β‐hydroxybutyrate concentrations were measured 30 min following each supplement intake (βHB, GlucoMen Areo 2 K‐meter with b‐ketone sensor strips, A. Menarini Diagnostics, Firenze, Italy). Furthermore, capillary acid–base balance, blood gasses, and glucose and lactate concentrations were measured using an ABL90 FLEX analyzer (Radiometer Medical ApS, Copenhagen, Denmark) before hypoxic entry (baseline), as well as before the onset of EX_MAX1_, EX_MAX2_, and EX_COMBO_. AMS symptoms were evaluated using a Lake Louise Score (LLS) questionnaire (Roach et al., 2018) before hypoxic entry, and after 4, 9, 11.5, 23, and 28.5 h in hypoxia.

### 2.6 Polysomnography (PSG)

A digital amplifier (V‐amp, Brain Products, Gilching, Germany) was used to record sleep quality and quantity during the experimental sessions, and data were digitized at a sampling rate of 1000 Hz. A blinded, independent, certified sleep technician (Sleep Well PSG, Canada) visually scored night recordings following the Rechtschaffen & Kales guidelines, in conjunction with the AASM guidelines, and all methodological details are identical to our earlier publications (Robberechts et al., 2023; Stalmans, Tominec, Robberechts, et al., 2025). The assessed sleep variables were: (i) total sleep time, (ii) wakefulness after sleep onset (WASO) and number of arousals, (iii) total N1, N2, rapid eye movement (REM) and slow wave sleep (SWS) duration, and (iv) sleep efficiency (total sleep time/time in bed).

### 2.7 Statistical analyses and sample size calculation

All statistical analyses were performed in GraphPad Prism version 9.3.1 (GraphPad Software, La Jolla, CA, USA). For data obtained at a single timepoint during each session (e.g., sleep efficiency, SWS duration, …), normality was tested using a Shapiro–Wilk test, and a paired *t*‐test or nonparametric Wilcoxon matched‐pairs signed rank test was used for normally distributed or skewed data, respectively. Data obtained at multiple timepoints during each session were analyzed using a two‐way repeated measures analysis of variance (ANOVA), or with the mixed‐effects model offered by GraphPad Prism in the case of missing data. Sphericity was evaluated using a Mauchly's test, and a Greenhouse–Geisser correction was applied when homoscedasticity was not met. Effect sizes (*η*_p_^2^ or hedges' g) were calculated only for significant effects, while post‐hoc analyses were only performed in case of significant interaction effects using Šidák's correction. Reported p‐values generally refer to the main effects or to these post‐hoc analyses in case of a significant interaction effect. Statistical significance was defined as *p* < 0.05, data in‐text are presented as mean ± SD unless otherwise specified. Data in Figures and violin plots are presented as median ± IQR. An a‐priori sample size calculation was performed for the larger project (Stalmans et al., 2024), indicating a required sample size of *n* = 10 per condition to identify an effect of KE intake on hypoxic tolerance and desaturation. Given that the present manuscript reports the data from 5 participants, the conclusions of the current manuscript should be considered exploratory. Nonetheless, we are confident that these data provide a valuable addition to our current knowledge on the effects of exogenous ketosis in hypoxic conditions.

## 3 RESULTS

### 3.1 Ketone ester intake induced ketosis without affecting acute mountain sickness symptoms

Throughout the diurnal parts of the protocol, KE intake increased blood [βHB] to 2–3 mM at all timepoints (range: 1.9–4.2 mM, *p* < 0.001 for time × KE effect, *η*_p_^2^ = 0.761, post‐hoc for KE vs. CON: *p* < 0.001) except upon waking up ([βHB] = 0.5 ± 0.2 mM, *p* > 0.999 for KE vs. CON, Figure 2a). In CON, [βHB] remained at 0.4–0.5 mM at all timepoints. Both total LLS and the LLS headache score showed a main time effect (*p* < 0.001 and *p* = 0.003, *η*_p_^2^ = 0.789 and *η*_p_^2^ = 0.695, Figure 2b,c, resp.). In both groups, total LLS increased after 9 h in hypoxia and remained elevated—yet below the protocol termination limit of 10—until the end of the protocol (*p* < 0.009 for all), while LLS headache scores increased after 9 h (*p* = 0.011) yet had returned to baseline after 28.5 h (*p* = 0.518).

**FIGURE 2:**
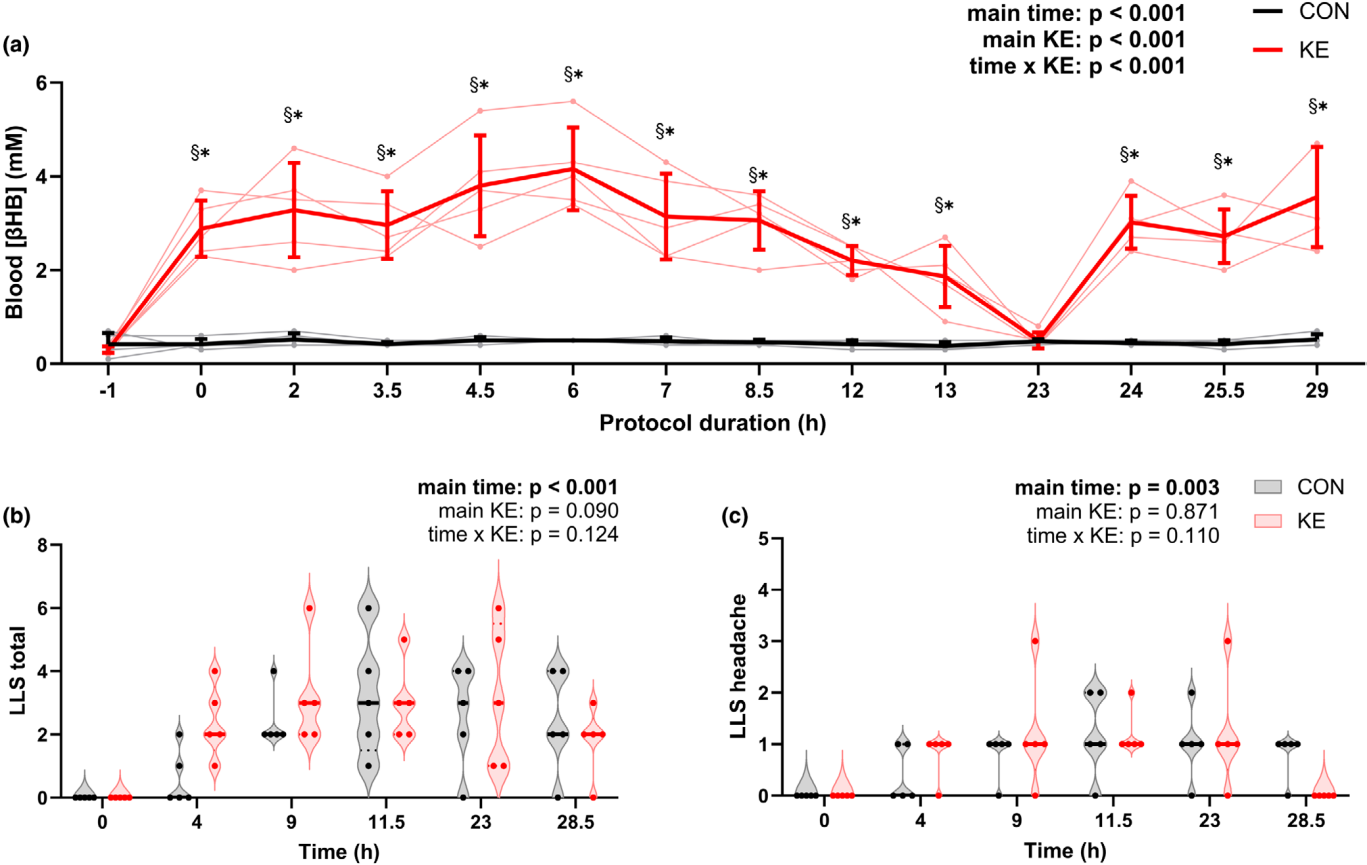
The effects of KE intake on blood β‐hydroxybutyrate and acute mountain sickness symptoms. Data are shown for (a) capillary blood β‐ hydroxybutyrate (βHB) concentrations, as well as (b) total and (c) headache scores of the Lake Louise Score (LLS) questionnaire. (a) Mean values (heavy lines) and SD (whiskers), as well as individual data (thin lines) are shown. (b, c) Data are presented as violin plots showing the distribution of values for each group, and median (full line) ± quartiles (dotted line) are shown. Ketone ester, KE; placebo session, CON. *p*‐values from a repeated‐measures ANOVA are printed for each graph. §, *p* < 0.05 vs. 15 min for indicated conditions; §, *p* < 0.05 vs. baseline for indicated condition; *, *p* < 0.05 for KE vs. CON.

### 3.2 Ketone ester intake prevented exercise performance declines

The achieved PPO was similar for EX_MAX1_ and EX_MAX2_ (main time effect: *p* = 0.065, Figure 3a) without difference between KE and CON (main KE effect: *p* = 0.818, time × KE effect: *p* = 0.778). PPO during EX_COMBO_ was 36 W higher in KE compared to CON (*p* = 0.018, hedges' g = 1.18, Figure 3b). V̇O_2_peak decreased by 7% from EX_MAX1_ to EX_MAX2_ in both KE and CON (main time effect: *p* < 0.001, *η*_p_^2^ = 0.605, Figure 3c), and KE did not affect V̇O_2_peak during EX_MAX1_, EX_MAX2_ (main KE effect: *p* = 0.191, time × KE effect: *p* = 0.162) or EX_COMBO_ (*p* = 0.340, Figure 3d). V̇_E_peak was similar between KE and CON for EX_MAX1_ and EX_MAX2_ (main time effect: *p* = 0.466, main KE effect: *p* = 0.703, time × KE effect: *p* = 0.868, Figure 3e) but 15% higher for KE vs. CON in EX_COMBO_ (*p* = 0.018, hedges' g = 0.977, Figure 3f).

**FIGURE 3:**
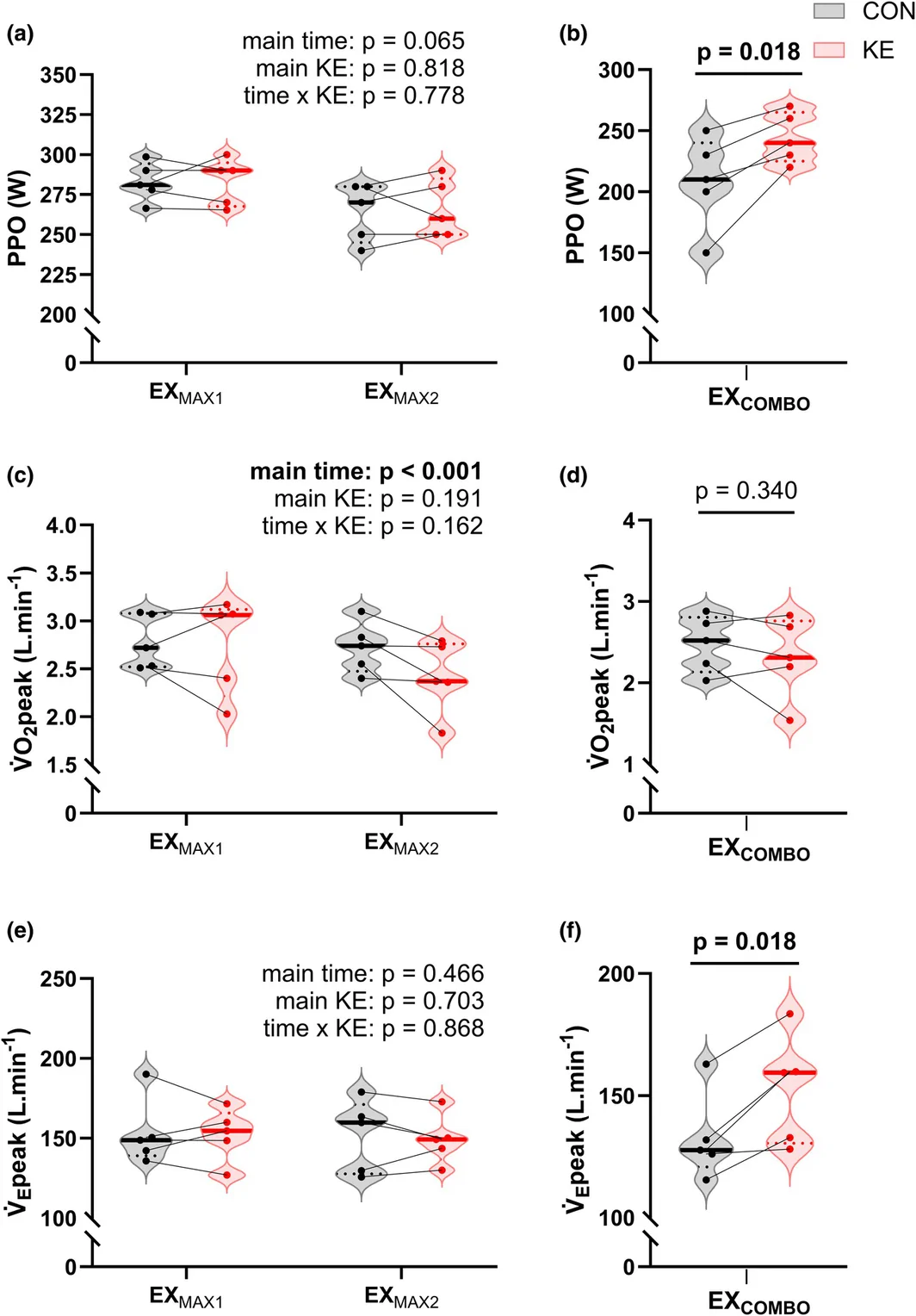
The effects of KE intake on exercise performance and physiology. Data are shown for (a, b) peak power output (PPO), (c, d) peak oxygen uptake rate (V̇O_2_peak), and e, f) peak ventilation (V̇_E_peak) during maximal exercise (panels a, c, e) and a combined exercise bout (panels b, d, f). Data are presented as violin plots showing the distribution of values for each group, and median (full line) ± quartiles (dotted line) are shown. Ketone ester, KE; placebo session, CON; maximal incremental exercise test after 1.5 and 25 h, EX_MAX1_ and EX_MAX2_, resp.; combined exercise bout after 26.5 h in hypoxia, EX_COMBO_. *p*‐values from a repeated‐measures ANOVA (panels a, c, e) or *t*‐test (panels b, d, f) are printed for each graph.

### 3.3 Ketone ester intake attenuated hypoxemia and increased tissue oxygenation

#### 3.3.1 Resting measurements

A time × KE effect (*p* = 0.032, *η*_p_^2^ = 0.464) revealed that KE intake attenuated the drop in resting SpO_2_ after 4 h (*p* = 0.049 for KE vs. CON) and 28 h (*p* = 0.005 for KE vs. CON) in hypoxia (Figure 4a). While KE intake did not affect resting mTOI (*p* = 0.095 for main KE effect, *p* = 0.134 for time × KE effect, Figure 4b), cTOI was higher in KE versus CON after 28 h in hypoxia (*p* = 0.009 for time × KE effect, *η*_p_^2^ = 0.550, *p* = 0.007 for KE vs. CON after 28 h, Figure 4c).

**FIGURE 4:**
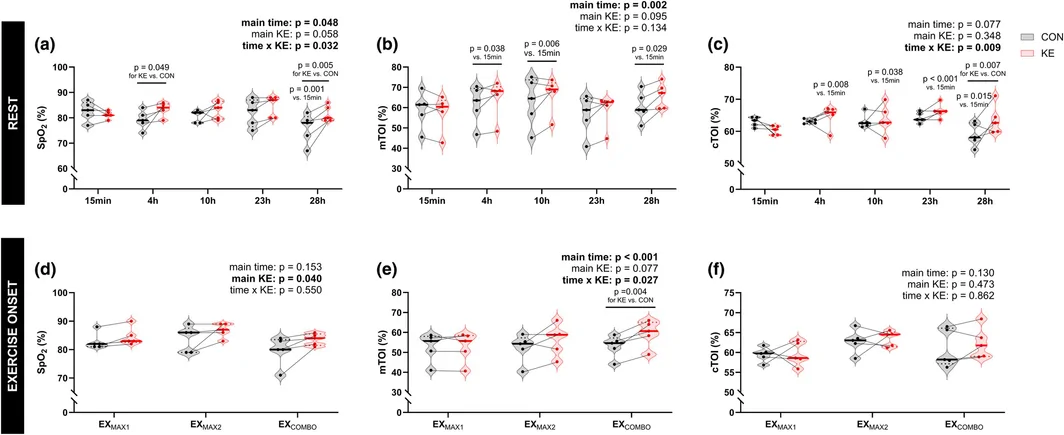
The effects of KE intake on oxygenation. Data are shown for (a–d), blood oxygen saturation (SpO_2_), (b–e) muscle tissue oxygenation index (mTOI), (c–f) cerebral tissue oxygenation index (cTOI) at rest (panel a–c) or in a seated position before onset of exercise (panel d–f). Data are presented as violin plots showing the distribution of values for each group, and median (full line) ± quartiles (dotted line) are shown. Ketone ester, KE; placebo session, CON; maximal incremental exercise test after 1.5 and 25 h, EX_MAX1_ and EX_MAX2_, resp.; combined exercise bout after 26.5 h in hypoxia, EX_COMBO_. *p*‐values from a repeated‐measures ANOVA are printed for each graph as well as significant post‐hoc *p*‐values.

#### 3.3.2 At the onset of maximal exercise

At the start of EX_MAX1_, EX_MAX2_, and EX_COMBO_, SpO_2_ was consistently elevated with KE (*p* = 0.040 for main KE effect, *η*_p_^2^ = 0.632, Figure 4d), although this did not reach significance post‐hoc for any exercise bout (*p* > 0.057 for KE vs. CON). While mTOI (*p* = 0.027 for time × KE effect, *η*_p_^2^ = 0.596, Figure 4e) did not increase with KE before the start of EX_MAX1_ and EX_MAX2_ (*p* = 0.999 and *p* = 0.053 for KE vs. CON, resp.), values were substantially elevated before the start of EX_COMBO_ (*p* = 0.004 for KE vs. CON). cTOI was similar in all conditions and at all timepoints (*p* > 0.130, Figure 4f).

### 3.4 Ketone ester intake induced acidosis and reduced pCO_2_ and bicarbonate concentrations

At the start of all maximal and combined exercise bouts, capillary pH, pCO_2_, and [HCO_3_^−^] were consistently reduced in KE versus CON (all *p* < 0.001 for main KE effect, *η*_p_^2^ = 0.943, *η*_p_^2^ = 0.942, *η*_p_^2^ = 0.966, Figure 5a–c, resp.). Conversely, KE elevated capillary pO_2_ at all timepoints (*p* = 0.0267 for main KE effect, *η*_p_^2^ = 0.795, Figure 5d). The parameter p50, as well as capillary [glucose] and [lactate] were not affected by KE (*p* > 0.238, *p* > 0.089, *p* > 0.223 for main KE or time × KE effects, Figure 5e–g, resp.).

**FIGURE 5:**
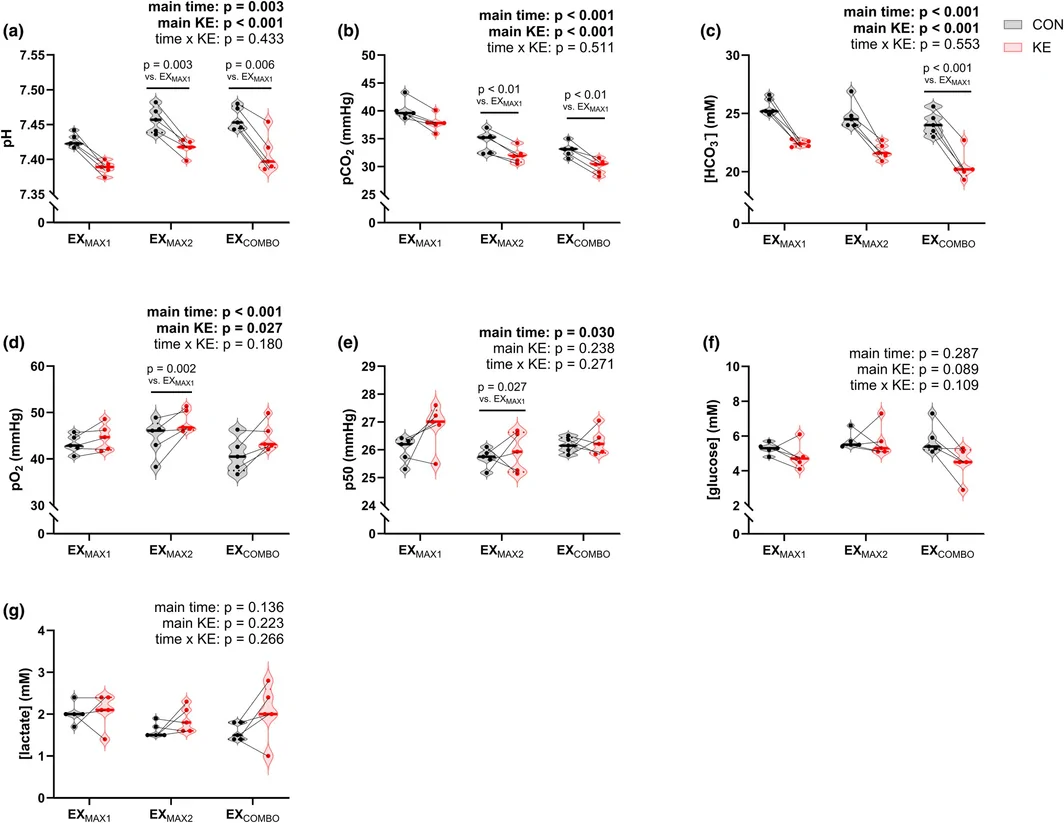
The effects of KE intake on capillary acid–base, blood gases, and metabolites. Data are shown for (a) blood pH, (b) partial pressure of CO_2_ (pCO_2_), (c) bicarbonate (HCO_3_^−^) concentrations, (d) partial pressure of oxygen (pO_2_), (e) the partial pressure of oxygen corresponding to a blood oxygen saturation of 50% (p50), (f), blood glucose concentrations, and (g) blood lactate concentrations measured before the start of exercise. Data are presented as violin plots showing the distribution of values for each group, and median (full line) ± quartiles (dotted line) are shown. Ketone ester, KE; placebo session, CON; maximal incremental exercise test after 1.5 and 25 h, EX_MAX1_ and EX_MAX2_, resp.; combined exercise bout after 26.5 h in hypoxia, EX_COMBO_. *p*‐values from a repeated‐measures ANOVA are printed for each graph as well as significant post‐hoc *p*‐values.

### 3.5 Ketone ester intake did not improve sleep in hypoxia

Intake of KE did not affect total sleep time (*p* = 0.742, Figure 6a), WASO (*p* = 0.813, Figure 6b), number of arousals (*p* = 0.164, Figure 6c), or sleep efficiency (*p* = 0.670, Figure 6d). Also the duration of the N1, N2, REM, and SWS phases was unaffected by KE (*p* = 0.698, *p* = 0.872, *p* = 0.392, and *p* = 0.702, Figure 6e–h, resp.). SpO_2_ and HR values during the night are shown in Table 1, and all parameters were similar in KE versus CON (all: *p* > 0.060).

**FIGURE 6:**
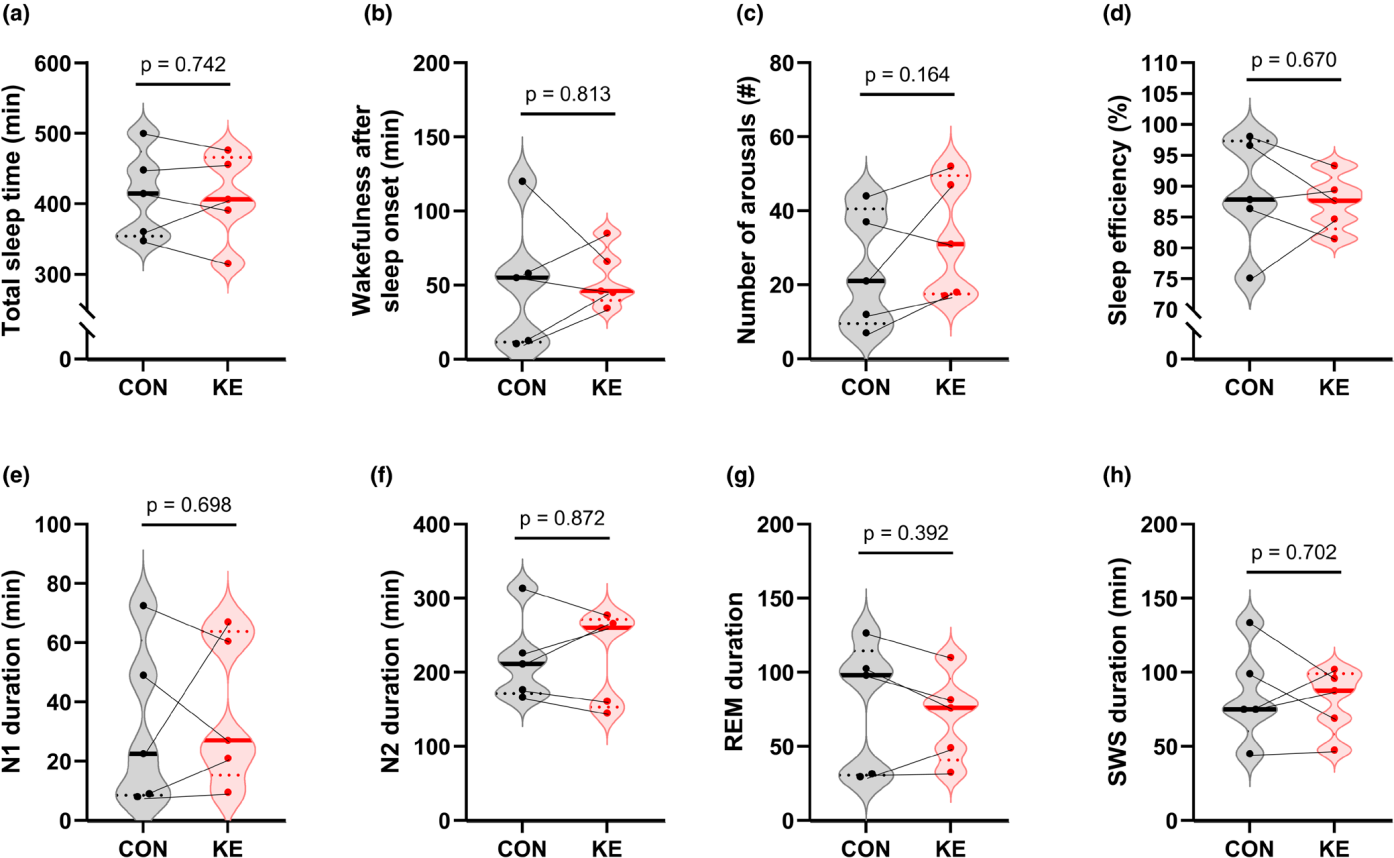
The effects of KE on sleep quality and architecture. Data are shown for (a), total sleep time, (b) wakefulness after sleep onset, (c) number of arousals during the night, (d) sleep efficiency, as well as the duration of the (e) N1, (f) N2, (g) rapid eye movement (REM), and (h) slow wave sleep (SWS) phases. Throughout the night, participants' sleep was recorded using polysomnography (PSG). Data are presented as violin plots showing the distribution of values for each group, and median (full line) ± quartiles (dotted line) are shown. Ketone ester, KE; placebo session, CON. *p*‐values from a paired *t*‐test are printed for each graph.

**TABLE 1:** Effect of ketone ester (KE) ingestion on nocturnal blood oxygen saturation and heart rate in hypoxia.

	CON	KE	*p*‐value
Blood oxygen saturation			
Average (%)	75 ± 6	76 ± 5	0.409
Minimum (%)	58 ± 10	63 ± 4	0.357
<90% (min)	418 ± 172	459 ± 65	0.875
<80% (min)	343 ± 189	356 ± 175	0.625
<70% (min)	98 ± 133	86 ± 113	0.625
<60% (min)	5 ± 10	0 ± 0	0.250
Heart rate			
Average (bpm)	65 ± 23	71 ± 19	0.060
Minimum (bpm)	49 ± 19	51 ± 16	0.324
>75 bpm (min)	232 ± 203	239 ± 226	0.700
>100 bpm (min)	10 ± 18	13 ± 24	0.309

*Note*: In a randomized, double‐blind, placebo‐controlled, crossover design study, participants performed two 29‐h sessions at a simulated altitude of 4000–4500 m, involving regular ketone ester (KE) or placebo (CON) ingestion. Throughout the night, participants' blood oxygen saturation and heart rate were monitored. Data are means ± SD for the subgroup (*n* = 5) of a larger project (Stalmans et al., 2024), that completed the full protocol in both sessions. Statistical differences between KE and CON were tested using a *t*‐test or Wilcoxon test and *p*‐values are shown.

### 3.6 Cognitive function was not affected by ketone ester intake or time in hypoxia

All cognitive parameters (Table 2) remained similar, without any effects of time in hypoxia (*p* > 0.092 for main time effects) or KE (*p* > 0.163 for main KE effects, *p* > 0.155 for time × KE effects).

**TABLE 2:** Effect of ketone ester (KE) ingestion on cognitive function in hypoxia.

	PRE EX_MAX1_	POST EX_MAX1_	5 h	8.5 h	PRE EX_MAX2_	POST EX_MAX2_	*p*‐values
RTI—MDMT (ms)							*t*: 0.794 KE: 0.228 txKE: 0.155
CON	154 ± 37	168 ± 31	167 ± 42	152 ± 31	149 ± 44	159 ± 43	
KE	144 ± 28	146 ± 36	143 ± 35	154 ± 52	153 ± 39	148 ± 36	
RTI—MDRT (ms)							*t*: 0.289 KE: 0.458 txKE: 0.434
CON	396 ± 45	415 ± 37	410 ± 37	425 ± 53	404 ± 57	411 ± 43	
KE	395 ± 47	391 ± 24	413 ± 36	415 ± 64	405 ± 53	399 ± 31	
RTI—TES (#)							*t*: 0.357 KE: 0.524 txKE: 0.554
CON	2 ± 2	1 ± 0	1 ± 1	1 ± 2	1 ± 1	1 ± 1	
KE	1 ± 1	1 ± 1	1 ± 1	1 ± 1	2 ± 0	1 ± 0	
RVP—MDL (ms)							*t*: 0.681 KE: 0.514 txKE: 0.480
CON	474 ± 43	488 ± 39	499 ± 34	502 ± 49	476 ± 79	483 ± 58	
KE	504 ± 95	495 ± 50	497 ± 54	488 ± 62	492 ± 70	487 ± 59	
RVP—TFA (#)							*t*: 0.092 KE: 0.163 txKE: 0.300
CON	0 ± 1	2 ± 2	2 ± 2	2 ± 1	1 ± 1	2 ± 2	
KE	1 ± 1	4 ± 2	1 ± 1	2 ± 2	3 ± 2	2 ± 1	
RVP—TH (#)							*t*: 0.316 KE: 0.739 txKE: 0.202
CON	41 ± 11	40 ± 13	44 ± 11	37 ± 13	40 ± 13	42 ± 15	
KE	41 ± 11	43 ± 10	39 ± 12	39 ± 14	40 ± 14	40 ± 15	
SWM—S							*t*: 0.588 KE: 0.780 txKE: 0.243
CON	3 ± 2	5 ± 2	3 ± 2	4 ± 1	3 ± 1	3 ± 2	
KE	5 ± 3	4 ± 2	4 ± 2	3 ± 1	4 ± 3	4 ± 1	
SWM—TE (#)							*t*: 0.501 KE: 0.613 txKE: 0.651
CON	3 ± 5	3 ± 5	1 ± 2	4 ± 7	4 ± 8	3 ± 6	
KE	4 ± 4	1 ± 1	2 ± 3	0 ± 0	5 ± 6	3 ± 6	

*Note*: In a randomized, double‐blind, placebo‐controlled, crossover design study, participants performed two 29‐h sessions at a simulated altitude of 4000–4500 m, involving regular ketone ester (KE) or placebo (CON) ingestion. Immediately before (PRE) and after (POST) the first and second maximal exercise tests (EX_MAX1_ and EX_MAX2_, resp.), as well as after 5 and 8.5 h in hypoxia, they performed a cognitive test battery to evaluate their reaction time (RTI), rapid visual information processing (RVP), and spatial working memory (SWM). Outcome parameters of each test were (i) RTI: median reaction time (RTI‐MRT), median movement time (RTI‐MMT), and number of errors out of 30 (RTI‐E); (ii) RVP: mean response latency (RVP‐MRL), number of correct responses (hits) out of 54 (RVP‐H), and false alarms out of 54 (RVP‐FA), and (iii) SWM: strategy score (SWM‐SS) and number of errors (SWM‐E). Data are means ± SD for the subgroup (*n* = 5) of a larger project (Stalmans et al., 2024), that completed the full protocol in both sessions. Statistical effects of time (*t*), ketone intake (KE), or their interaction (txKE) were tested using a mixed‐effects model offered by Graphpad Prism, and *p*‐values are shown for the main and interaction effects.

## 4 DISCUSSION

This manuscript presents the data obtained from a subgroup of five non‐AMS developing participants who participated in a larger project (Stalmans et al., 2024). While the present study is likely underpowered for some outcomes due to the limited sample size, we found that KE intake did not affect PPO achieved during EX_MAX1_ or EX_MAX2_ at a simulated altitude of 4000 m. Conversely, KE increased PPO during EX_COMBO_ (i.e., a maximal incremental exercise test performed immediately following 30 min cycling at 1.5 W·kg^−1^, performed after 26.5 h in hypoxia), accompanied by increased blood and muscle oxygenation before the onset of EX_COMBO_. Furthermore, KE intake increased resting blood, muscle and brain oxygenation at rest during the diurnal parts of the protocol, but did not impact sleep quality, nocturnal heart rate or blood oxygenation, or cognitive function.

Our data indicate that PPO achieved during EX_MAX1_ and EX_MAX2_ was unaffected by KE, despite reduced HCO_3_^−^ concentrations with KE before the start of both. This is consistent with previous research (Poffe et al., 2021), in which the mean power output during a 15‐min time trial performed at the end of a 3‐h race simulation under hypoxic conditions was not reduced by KE intake, likely due to a concomitant increase in blood and skeletal muscle oxygenation induced by KE. In the current study, SpO_2_ was also increased by KE at the start of EX_MAX1_ and EX_MAX2_. This ketone‐induced ‘hyper‐oxygenation’ is detailed in our earlier publication (Stalmans, Tominec, Lauriks, et al., 2025), and relates to a combination of ketone‐induced acidosis and the sigmoidal nature of the oxyhemoglobin dissociation curve (ODC). In short, ketone‐induced acidosis stimulates ventilation, causing an increase in blood pO_2_ that translates to increased SpO_2_ when an individual's steady‐state resides within the ‘steep’ part of the ODC. As such, the primary factor to negate the ergolytic effect of KE in hypoxia appears to be blood oxygenation.

While KE intake did not affect performance during EX_MAX1_ and EX_MAX2_, it increased PPO achieved during EX_COMBO_, which consisted of 30 min low‐intensity exercise (1.5 W·kg^−1^) followed by an identical ramp protocol. This beneficial effect may either result from (i) better recovery between EX_MAX2_ and EX_COMBO_, and/or (ii) direct ergogenic responses during EX_COMBO_. With regard to the first, we recently observed that exogenous ketosis induced by (R)‐1,3 butanediol ingestion increased post‐exercise blood flow through the common femoral artery, both at sea level and simulated altitude (3000 m) (Stalmans, Narang, et al., 2026). In the same study, exogenous ketosis elevated microvascular function, evidenced by increased skeletal muscle reoxygenation rates following 5 min of vascular occlusion. With regard to direct ergogenic effects, KE has been proposed to stimulate post‐exercise glycogen resynthesis (Holdsworth et al., 2017) and to mediate glycogen‐sparing effects (Cox et al., 2016), although both hypotheses have also been rightfully questioned or even rejected (Poffé et al., 2020; Vandoorne et al., 2017). Moreover, mTOI was higher with KE at the start of EX_COMBO_, but not EX_MAX1_ and EX_MAX2_, suggesting that muscular oxygenation may be a critical factor in the beneficial effect of KE. An interesting observation is that the higher PPO values achieved during EX_COMBO_ were not accompanied by higher V̇O_2_peak values, suggesting that ketone bodies may increase whole‐body oxygen‐efficiency. While our data do not allow to confirm this hypothesis, this is in line with two recent studies reporting increased efficiency following KE intake in the absence of carbohydrate ingestion (Brady & Egan, 2024; Dearlove et al., 2021).

Hypoxia‐induced sleep disruptions may compromise both exercise performance and post‐exercise recovery (Blacker et al., 2021; Chase et al., 2017; Malle et al., 2013; Steinman et al., 2023). However, KE intake did not affect any sleep‐related parameters in the current study. We previously identified that KE intake counteracted the drop in sleep efficiency following strenuous, late‐evening exercise under normoxic conditions (Robberechts et al., 2023), but did not attenuate reductions in sleep efficiency during acute exposure to a simulated altitude of 3000 m (Stalmans, Tominec, Robberechts, et al., 2025). These findings indicated that KE only appeared effective when REM sleep was disrupted through, for instance, late evening exercise, but not when NREM sleep was disrupted, as is generally the case with hypoxic exposure (Stalmans, Tominec, Robberechts, et al., 2025).

Finally, our data showed that KE intake does not improve cognitive performance in non‐AMS developing individuals in hypoxia, which is in line with the data from the larger project (Stalmans, Tominec, Lauriks, et al., 2025). On the contrary, the work of McClure et al. (McClure, Phillips, Koutnik, et al., 2024) reported beneficial effects of exogenous ketosis on cognitive performance at higher altitudes (~6100 m), where ketone monoester intake attenuated the decline in cognitive efficiency during a Defense Automated Neurobehavioral Assessment. Of note, our protocol does not allow us to identify if hypoxia per se impacted cognitive function. Therefore, we acknowledge the possibility that exogenous ketosis may still prevent cognitive decline in the face of hypoxia, without improving undisrupted cognitive function.

### 4.1 Limitations

Evaluating the data from the non‐AMS developing subgroup (*n* = 5) from our earlier project (Stalmans et al., 2024) uniquely allows to explore the effects of KE intake during rest and exercise at a relatively high altitude (4000 m) without the confounding effect induced by AMS symptoms. Nonetheless, the selection for individuals who do not develop severe AMS may compromise its translatability to a broader public, particularly given the estimated 50%–80% incidence of AMS in a general population at altitudes of 4200–4500 m (Beidleman et al., 2013; Hackett et al., 1976; Mairer et al., 2009). Moreover, the small sample size (*n* = 5) substantially lowered the power of the study, thereby increasing the risk for false negative findings. Nonetheless, we are confident that the reported significant results accurately reflect physiological responses, thereby contributing to our current understanding of the effects of exogenous ketosis on endurance performance, sleep, and cognitive function under hypoxic conditions. Another important consideration is that only males were recruited for this study given the ambiguous role of the hormonal cycle on endurance (Carmichael et al., 2021) and cognitive (Le et al., 2020) performance, as well as the impact of menstruation‐related discomfort on AMS scoring (Stalmans et al., 2024). Therefore, it remains to be identified whether similar effects would also be observed in female individuals. Another important consideration is the caloric difference between the non‐caloric placebo and the KE drinks. As such, we cannot exclude that the observed differences on performance are in part related to energy intake. It is, however, unlikely that this may have provoked the observed effects given that all participants received a significant caloric surplus (i.e., 4000 kcal over the 29‐h protocol) [56]. Moreover, employing an isocaloric placebo containing either carbohydrates or fats would considerably alter substrate metabolism, supporting our decision to use an inert placebo. Similarly, the mode of exogenous ketone administration substantially impacts the subsequent physiological landscape. For instance, with KE intake, it is impossible to isolate the direct effects from ketone bodies from the accompanying effects of changes in acid–base balance, metabolism, or substrate utilization. Finally, normoxic baseline values were not evaluated in the protocol, which prevents evaluating the effects of hypoxia per se. We also wish to highlight that the findings from this study, acute in nature, may not be readily extrapolatable to chronic setting. No data were obtained regarding liver biochemistry. Although such insights would have added great value, our primary aim was to evaluate the effects of KE during hypoxic exposure.

## 5 CONCLUSIONS

Altogether, we found that KE intake appears effective in attenuating declines in high‐intensity endurance performance for non‐AMS developing individuals during subacute hypoxic exposure, involving repeated exercise until exhaustion. While it remains to be determined whether this effect results from direct ergogenic effects or enhanced post‐exercise recovery, these data reveal an interesting perspective for the use of ketones during multi‐day, high‐altitude events, as frequently encountered by ultra‐endurance runners and mountaineers, as well as mountain rescue staff. Finally, diverse patient populations (e.g., COPD, pulmonary hypertension) encounter physiological fingerprints that strongly resemble the effects of hypoxic exposure. Therefore, it would be interesting to identify if any performance benefits elicited by KE intake could potentially translate into higher functional capacities and, eventually, quality of life in patient populations.

## AUTHOR CONTRIBUTIONS

**Myrthe Stalmans:** Conceptualization; data curation; formal analysis; funding acquisition; investigation; methodology; project administration; resources; software; supervision; validation; visualization. **Domen Tominec:** Data curation; investigation. **Wout Lauriks:** Data curation; investigation. **Ruben Robberechts:** Data curation; investigation. **Monique Ramaekers:** Data curation; investigation. **Tadej Debevec:** Conceptualization; data curation; funding acquisition; investigation; methodology; project administration; resources; supervision. **Chiel Poffé:** Conceptualization; data curation; formal analysis; funding acquisition; investigation; methodology; project administration; resources; software; supervision; validation; visualization.

## FUNDING INFORMATION

This research was supported by the Research Foundation—Flanders (FWO Weave, research grant G073522N) and Slovene Research Agency grant (N5‐0247). CP is supported by an FWO postdoctoral research grant (12B0E24N).

## CONFLICT OF INTEREST STATEMENT

The authors declare that they have no competing interests.

## ETHICS STATEMENT

This project was approved by the Ethics Committee Research UZ/KU Leuven (B3222022000810), preregistered at www.clinicaltrials.gov (NCT05588427), and conducted in accordance with the Declaration of Helsinki guidelines.

## Supporting information


**Figure S1.** Individual datapoints (*n* = 14) showing participants' tolerated time in the hypoxic protocol in response to regular ingestion of ketone ester (KE, red bars) or placebo (CON, black bars) drinks during a 29‐h normobaric hypoxic protocol. Individuals' KE and CON sessions are visualized side by side. Each bar displays the main withdrawal reasons, defined as a score of ≥2 out of 3 on the different criteria of the validated Lake Louise Score questionnaire (Roach et al., 2018); D, Dizziness or light‐headedness; F, Fatigue or weakness; H, headache; N, nausea or gastrointestinal symptoms.

## Data Availability

Data will be made available upon reasonable request.
